# Association Between Depression, Anxiety, Quality of Life and Burnout Syndrome with Symptoms of Insomnia in Healthcare Professionals in Montenegro During the COVID-19 Pandemic

**DOI:** 10.3390/jcm14103374

**Published:** 2025-05-12

**Authors:** Dragana Backović, Dragana Jovanović, Ana Tomas, Zoran Bukumirić, Kristina Tomović

**Affiliations:** 1 Clinical Center of Montenegro, 81000 Podgorica, Montenegro; b.dragana@ucg.ac.me (D.B.); dragana.j@ucg.ac.me (D.J.); 2 Faculty of Medicine, University of Montenegro, 81000 Podgorica, Montenegro; 3 Department of Pharmacology and Toxicology, Faculty of Medicine Novi Sad, University of Novi Sad, 21000 Novi Sad, Serbia; 4 Institute of Medical Statistics and Informatics, Faculty of Medicine, University of Belgrade, 11080 Belgrade, Serbia; zoran.bukumiric@med.bg.ac.rs; 5 Primary Healthcare Center Podgorica, 81000 Podgorica, Montenegro; domzdravljapgd@t-com.me

**Keywords:** insomnia, burnout syndrome, crisis, healthcare professionals, occupational stress

## Abstract

**Background**: The COVID-19 pandemic significantly impacted the mental health and well-being of healthcare professionals worldwide. This study investigated the association between mental health factors, burnout syndrome, quality of life, and insomnia symptoms in healthcare professionals during the COVID-19 pandemic in Montenegro. **Methods**: A cross-sectional study was conducted between July and October 2021 among 299 healthcare professionals at the Clinical Center of Montenegro. Participants completed standardized questionnaires, including the Maslach Burnout Inventory (MBI-HSS), Athens Insomnia Scale (AIS), depression, anxiety, and stress scale (DASS-21), and EQ-5D health-related quality of life questionnaire. **Results**: Insomnia was reported in 65.0% of female and 35.0% of male participants, with a mean age of 38.57 ± 11.57 years. Insomnia symptoms were more common among those reporting alcohol consumption (*p* = 0.007), smoking (*p* = 0.006), and sedative use (*p* = 0.038). A higher workload (*p* = 0.017), previous COVID-19 infection (*p* = 0.001), and quarantine (*p* = 0.008) were linked to insomnia. Healthcare professionals with insomnia reported lower quality of life across all EQ-5D dimensions (*p* < 0.001) and higher levels of stress, anxiety, and depression (*p* < 0.001). Burnout was significantly associated with emotional exhaustion (*p* < 0.001), while depersonalization and personal achievement showed no significant differences. **Conclusions**: This study highlights a significant relationship between burnout, mental health issues, and insomnia during the COVID-19 pandemic. Addressing these factors through targeted interventions and workplace policies is essential for improving healthcare professionals’ well-being and ensuring the healthcare system’s sustainability.

## 1. Introduction

The coronavirus disease (COVID-19) had a profound impact on various aspects of society [1,2,3,4]. It disrupted global economies, altered social interactions, and challenged healthcare systems. Healthcare professionals (HCPs) were particularly affected, with increased reports of depression and stress in this population [5]. They faced longer working hours and experienced a high risk of exposure, coupled with the emotional toll of caring for critically ill patients. Burnout syndrome, which is common among doctors, can occur early in an individual’s medical career [6], with reported prevalence among medical students being between 40% and 76% [7] linked to academic pressure, financial stress, and the physical and emotional toll of clinical rotations. Nurses, maintaining close and prolonged patient contact, are especially vulnerable due to physical demands, staffing shortages, and high patient loads [8]. The COVID-19 pandemic made working conditions even harder, with nurses working extended shifts. Burnout’s effects extend beyond the workplace, leading to emotional exhaustion, loneliness, depersonalization, and the deterioration of personal relationships [6]. HCPs experiencing burnout may struggle to maintain a work–life balance, withdraw socially, and experience significant personal struggles. Addressing instances of burnout is crucial to protect the mental health of HCPs and ensure the quality of patient care.

Sleep disorders, particularly insomnia, have become common in modern society, with significant implications for physical and mental health, affecting a person’s overall quality of life. Chronic insomnia is associated with a range of health issues, including cardiovascular disease, obesity, diabetes, and mental health disorders [9,10,11]. The prevalence of sleep disorders has been on the rise in modern society due to different factors, including increased screen time, irregular work schedules, and stress levels in contemporary life [12]. Healthcare professionals are particularly susceptible to insomnia due to occupational stress and demanding, often irregular, work schedules. The nature of healthcare work often involves shift work, on-call duties, and the need to make critical decisions under pressure, all of which can disrupt normal sleep patterns [13,14]. Additionally, the emotional intensity of patient care can lead to anxiety, further impacting sleep quality [15].

As mentioned before, the pandemic has subjected healthcare workers to increased workloads and emotional distress [16]. What is more, the rapidly evolving nature of the pandemic has required healthcare professionals to constantly adapt to new protocols and information, contributing to cognitive overload and difficulty in “switching off” after work [17]. This has resulted in heightened psychological distress and sleep disturbances among this population. Studies have shown increased rates of insomnia, anxiety, and depression among healthcare workers during the pandemic [17,18]. The combination of physical exhaustion, emotional strain, and disrupted routines contributes to sleep disorders [19]. A survey of 2355 Italian nurses found a high prevalence of insomnia (65.4%) among night-shift nurses, highlighting the need for further research into the risk factors contributing to this significant occupational health issue [20]. Given the significant impact of sleep disorders on overall well-being and job performance, it is important to investigate the factors associated with insomnia in healthcare professionals [21]. Poor sleep quality can lead to decreased cognitive function and impaired decision-making and cause an increased risk of medical errors [22]. It can also contribute to burnout and negatively impact the quality of patient care [16].

Data on the impact of the COVID-19 pandemic on the well-being of HCPs in Montenegro include reports of an increase in lumbar pain and an increase in burnout prevalence in this population [23,24]. In Montenegro, there are specific challenges faced by healthcare workers in this region, as there are unique cultural, economic, and healthcare system factors that could influence sleep patterns and well-being. This study aimed to examine the relationship between burnout, occupational factors, COVID-19 exposure, mental health, and health-related quality of life with symptoms of insomnia among healthcare professionals in Montenegro.

## 2. Materials and Methods

### 2.1. Setting and Sample Description

This cross-sectional study was conducted between July and October 2021 at the Clinical Center of Montenegro (CCM), Podgorica, Montenegro. The study was conducted in accordance with the Declaration of Helsinki and approved by the Ethical Committee of the Clinical Centre of Montenegro (No. 03/01-10292/1). Montenegro, a Southeastern European country, has a model universal healthcare system funded by mandatory health insurance contributions. Healthcare functions through primary healthcare centers, hospitals, and specialized healthcare facilities. The CCM is a hospital with more than 750 beds, a scientific and research center, and serves as a teaching base. It is the only tertiary healthcare medical institution in Montenegro conducting consultative specialist and subspecialist services. All HCPs who were employed full-time at the Clinical Center of Montenegro and participated in the treatment of patients suffering from COVID-19 were eligible for inclusion. This included all nurses, doctors, and other medical staff. During the study period, all wards were visited, and all HCPs present were included in the study.

### 2.2. Data Collection

For the purposes of this research, a general questionnaire was specially designed, and five validated and culturally adapted questionnaires—the general questionnaire for collecting socio-demographic data; the questionnaire for the assessment of burnout syndrome at work—the Maslach Burnout Inventory; the AIS—ATHENS INSOMNIA SCALE; the DASS scale—depression, anxiety, and stress scale; and EQ-5D, which is a questionnaire for measuring health-related quality of life—were used. The data collection instruments used are available as a Supplementary File S1.

#### 2.2.1. General Questionnaire

The general questionnaire had 23 questions. The first eight questions were related to the following socio-demographic characteristics: gender, age, marital status, number of children, habits (use of cigarettes, alcohol, sedatives), occupation, and the education of the respondents. Seven questions were related to data on the work unit, years of working experience, shift and overtime work, and the type of employment. In the remaining eight questions, respondents were surveyed regarding their work with COVID-19 patients during the pandemic, including the type and length of engagement, perceptions of feeling safe at work, the impact of the pandemic on employment and financial conditions, staying in quarantine, and COVID-19 vaccination status.

#### 2.2.2. The Athens Insomnia Scale

The Athens Insomnia Scale was used to assess the severity of insomnia using diagnostic criteria determined by the International Classification of Diseases-10 (ICD-10). The AIS was developed by Dr. Constantine Soldatos and colleagues [25,26]. The questionnaire consists of 8 questions that assess the onset of sleep, nighttime and early-morning awakenings, sleep time, sleep quality, the frequency and duration of complaints, and complaints caused by insomnia and disturbances in daily functioning. In the assessment of insomnia, a four-point Likert scale is used, where the respondents estimate how much sleep difficulties have affected their daily life in the past month. The degree of impact ranges from 0 (the specified item did not cause problems) to 3 (the specified item had a significant impact). The limit value of the scale is 6 [25,26].

#### 2.2.3. Health-Related Quality of Life EQ-5D-5L

The 5-level EQ-5D version (EQ-5D-5L, EuroQol Research Foundation, EuroQol Group, Rotterdam, The Netherlands) is a generic questionnaire that examines health-related quality of life. Each of the five dimensions of this questionnaire (mobility, self-care, usual activities, pain/discomfort, and anxiety/depression) has five levels: no problems, slight problems, moderate problems, severe problems, and extreme problems. Health score values range from 0 to 100, with 100 being the maximum value. At the same time, the value of the EQ-5D index can have a maximum value of 1, which reflects full health and quality of life.

#### 2.2.4. DASS Scale—The Depression, Anxiety, and Stress Scale

The DASS scale—the depression, anxiety, and stress scale—consists of 21 items that assess the intensity of three negative emotional states: depression, anxiety, and stress. It was developed by Sydney Lovibond & Peter Lovibond at the School of Psychology, University of New South Wales, Sydney, Australia. The instrument uses a four-point Likert scale, with the rating as follows—0: Did not apply to me at all; 1: Applied to me to some degree or some of the time; 2: Applied to me to a considerable degree or a good part of the time; and 3: Applied to me very much or most of the time. A higher score in each of the components indicates the severity of depression, anxiety, and stress [27].

#### 2.2.5. Maslach Burnout Inventory—Human Services Survey

For research purposes, the right to use the Maslach Burnout Inventory—Human Services Survey, MBI-HSS [28], for employees in institutions who are in direct contact with people (Human Services Survey, MBI-HSS) was secured from the developer (Mind Garden, Redwood City, CA, USA). This instrument is recognized as a leading measure of burnout and consists of a total of 22 items, based on which three aspects of burnout at work are assessed: the scale of emotional exhaustion EE (9 items), the DP depersonalization scale (5 items), and PA personal accomplishment scale (8 items). To assess each of the 3 elements of the burnout syndrome, a series of statements was given for which the respondent expressed the degree of agreement through a six-point Likert scale (0—never; 1—at least a few times a year; 2—at least once a month; 3—several times a month; 4—once a week; 5—several times a week; 6—every day). The limit values of the scales are as follows: the scale of emotional exhaustion: high EE ≥ 27 points, moderate EE = 17–26 points, and low EE ≤ 16; the depersonalization scale: high level of DP ≥ 13, moderate level of DP = 7–12, and low level of DP ≤ 6; and the scale of personal accomplishment: high level of PA ≥ 39, moderate level of PA = 32–38, and low level of PA ≤ 31. A high score for exhaustion and depersonalization contributes to burnout syndrome, while a high score for professional fulfillment reduces it [28].

### 2.3. Statistical Analyses

All data were processed in the IBM SPSS Statistics 22.0 (SPSS Inc., Chicago, IL, USA) software package. Depending on the type of variables and the normality of the distribution, the data description is shown as *n* (%) or the arithmetic mean ± standard deviation. Among the methods for testing statistical hypotheses, the following were used: the *t*-test, Mann–Whitney test, chi-square test, and Fisher’s exact probability test. The correlation between the variables was estimated using Spearman’s correlation coefficient (rho). Statistical hypotheses were tested at a statistical significance level (alpha level) of 0.05. Based on the sample sizes achieved, the study had over 90% power to detect small-to-medium effect sizes (Cohen’s h ≈ 0.3) for between-group comparisons, assuming a two-sided alpha of 0.05.

### 2.4. Use of Generative AI

We used ChatGPT to check for reference style formatting and Grammarly to check spelling and grammar.

## 3. Results

Out of the 300 questionnaires distributed, 299 were fully completed (response rate 99.7%). Of the participants included, 106 (35.5%) were male and 193 (64.5%) were female. The average age was 37.7 ± 11.4 years (range 18–65). Regarding the occupation, 83 participants (27.8%) were physicians, 160 (53.5%) were nurses/medical technicians, and 56 (18.7%) were from other medical professions. Among the 299 respondents included (doctors, medical technicians, and other medical staff), insomnia was present in 142 (47.5%) of respondents. The analysis of demographic data (Table 1) indicates no significant differences in sex, age, marital status, or the number of children between those with and without insomnia. However, lifestyle factors such as alcohol consumption (*p* = 0.007), cigarette use (*p* = 0.006), and sedative intake (*p* = 0.038) show statistically significant differences, with higher rates among individuals with insomnia. Work-related variables, including occupation, shift work, and overtime, did not show significant associations with insomnia (Table 1).

Individuals with insomnia were more likely to report high workloads and excessive engagement (*p* = 0.017). Additionally, undergoing quarantine during the pandemic (*p* = 0.008) and having a previous COVID-19 infection (*p* = 0.001) were significantly associated with insomnia (Table 2). COVID-19 engagement was significantly and more frequently reported in respondents with insomnia (82.2%); they were engaged with COVID-19 patients for more than 3 months (71.3%) and had a more commonly reported increase in workload and excessive engagement compared to respondents without insomnia. COVID-19 infection and quarantine were also more common in respondents with insomnia (Table 2).

### 3.1. Insomnia and Health-Related Quality of Life

Self-perceived health-related quality of life (Table 3) was notably lower among individuals with insomnia, with significant differences observed in mobility (*p* = 0.000), self-care (*p* = 0.000), usual activities (*p* = 0.000), pain/discomfort (*p* = 0.000), and anxiety/depression (*p* = 0.000). Respondents with insomnia had significantly lower scores for the self-assessment of health status (73.82 ± 18.799 vs. 89.26 ± 11.949), as well as significantly lower values of the EQ-5D index (0.89 ± 0.13 vs. 0.97 ± 0.85) (Table 3).

### 3.2. Insomnia and Mental Health Assessment

Mental health assessments (Table 4) using the DASS-21 scale showed higher levels of stress, anxiety, and depression in individuals with insomnia (*p* < 0.001 for all three measures). In respondents with insomnia, a significantly higher degree of stress (38.3% vs. 19%), a higher degree of anxiety (50.3% vs. 27.4%), and a higher degree of depression (38.2% vs. 17.6%) were observed in comparison to subjects without insomnia.

### 3.3. Insomnia and Burnout Assessment

**Figure 1:**
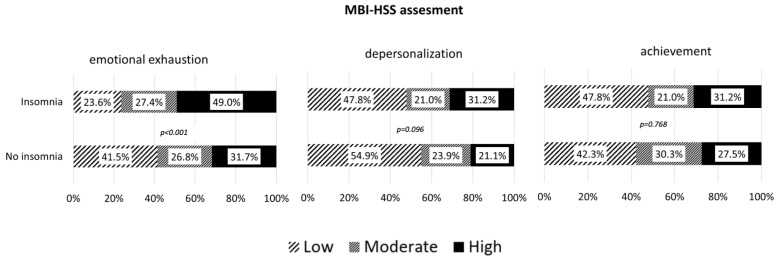
MBI-HSS assessment: degrees of emotional exhaustion, depersonalization, and accomplishment in respondents with and without insomnia.

Burnout assessments (Figure 1) demonstrated that emotional exhaustion was significantly higher among those experiencing insomnia (*p* < 0.001). A high degree of emotional exhaustion was more commonly recorded in subjects with insomnia (49.0% vs. 31.7%). Depersonalization (*p* = 0.096) and personal accomplishment (*p* = 0.768) did not show significant differences.

For the correlation analysis of the AIS total score and individual elements of the MBI scores, a statistically significant weak positive association between the MBI scores of emotional exhaustion (rho = 0.202; *p* < 0.001), depersonalization (rho = 0.125; *p* = 0.031) and the total AIS scores was found. There was no statistically significant correlation between the MBI scores for accomplishments and total AIS scores (rho = 0.020; *p* = 0.730).

## 4. Discussion

This cross-sectional study investigated the association between symptoms of insomnia and depression, anxiety, quality of life, and burnout syndrome among HCPs in Montenegro during the COVID-19 pandemic. Using validated and culturally adapted instruments, including the Maslach Burnout Inventory, Athens Insomnia Scale, DASS-21, and EQ-5D questionnaire, we assessed a comprehensive set of psychological and occupational factors. The findings of the present study reveal a high prevalence of insomnia symptoms among HCPs in Montenegro, with significant associations observed between insomnia symptoms and higher levels of depression, anxiety, stress, emotional exhaustion, as well as lower quality of life scores. These results highlight the considerable psychological burden faced by frontline healthcare providers during the pandemic.

Insomnia can lead to the disruption of the functioning of HCPs with public health consequences as the quality of the services provided affects the outcome of patient care. Lessons learned during the pandemic can support healthcare functioning in future crises and help guide decision-makers in implementing measures to reduce the onset of these symptoms and improve the overall quality of care. In the present study, conducted during the COVID-19 pandemic in Montenegro, insomnia symptoms were present in 142 (47.5%) of 299 surveyed HCPs. This is in line with previous studies, as the results of the umbrella review of meta-analyses showed that the prevalence of insomnia among HCPs during the COVID-19 pandemic was 36.36%, with reported prevalence ranging from 27.8% to 47.3 determined in the previous meta-analyses included [29]. In the present study, work-related variables (occupation, shift work, and overtime) were not significantly linked to insomnia, but lifestyle factors, such as alcohol, cigarette, and sedative use, were more commonly reported among HCPs with insomnia. The relationship between insomnia symptoms and the use of substances such as alcohol, cigarettes, and sedatives is a multifaceted issue. In the present study, the mental health assessment (DASS-21) revealed significantly higher stress (38.3% vs. 19%), anxiety (50.3% vs. 27.4%), and depression (38.2% vs. 17.6%) among individuals with insomnia (*p* = 0.000). The presence of insomnia, either as a standalone condition or together with other mental health challenges, may lead to maladaptive coping mechanisms, such as resorting to smoking and alcohol, which are substances that are readily accessible. Higher sedative use could also be directly related to insomnia and other mental health conditions. What is more, substance use can also exacerbate insomnia symptoms, creating a vicious cycle of dependence and sleep disruption [30]. An umbrella review by Sahebi et al. showed that the prevalences of anxiety and depression among HCWs were 24.92% and 21.41%, respectively [29], and the results of other studies also show that HCPs during COVID-19 were prone to psychiatric disorders [31,32].

This is the second part of the research conducted at the Clinical Center of Montenegro, where the first part focused on burnout syndrome, found in 14% of respondents, with the highest degree of emotional exhaustion being of particular concern, with almost two-thirds of respondents having a moderate or high level of emotional exhaustion [23]. According to the results of available studies on the connection between insomnia and burnout syndrome, there is no complete agreement. Although it is to be expected that exposure to stress with the exhaustion of compensatory mechanisms and the occurrence of burnout syndrome will lead to sleep disorders, the study by Metlaine et al. found no connection between insomnia and the occurrence of burnout syndrome [33]. On the other hand, in the study by Medisauskaite and Kamau in a population of physicians with burnout syndrome, 20–61% reported sleep problems, while 12% had severe/moderate insomnia [34]. Although the values of the individual elements of the DASS21 score were mostly within the limits of physiological values, the analysis and connection of these elements to those of the MBI score showed a correlation between depression, anxiety, stress, and burnout syndrome. Both during epidemics and pandemics, it has been observed that both the general population and HCPs experience an increase in stress and anxiety levels [21,35]. Some authors go so far as to question whether depression and burnout syndrome are two faces of the same disorder [36].

The COVID-19 pandemic has extended far beyond an immediate respiratory illness, affecting human existence and driving a surge in mental health disorders, including insomnia. In the present study, COVID-19 exposure significantly influenced insomnia: those with insomnia also reported higher rates of COVID-19 work engagement, longer work durations, being in quarantine, and past COVID-19 infection. A study involving 1705 frontline nurses and licensed practical nurses in Quebec, Canada, reported chronic fatigue, poor quality of care, lower work satisfaction, and a higher intention to leave the nursing organization caring for COVID-19 patients [37]. The vulnerability of healthcare workers to mental health issues, including insomnia, during the COVID-19 pandemic, has been well described, with the prevalence of insomnia being higher in HCPs with a higher prevalence of COVID-19-related work engagement, coupled with a higher workload [38]. Increased patient loads, uncertainty surrounding the disease, and fear of infection collectively contributed to work-related stress, anxiety, and burnout, all of which can trigger insomnia [19,39,40,41].

Insomnia not only disrupts nighttime rest but also daytime functioning, cognitive performance, emotional regulation, and overall well-being [42]. In HCPs in Montenegro, insomnia negatively impacted health-related quality of life (EQ-5D-5L), with significant impairments in mobility, self-care, usual activities, pain/discomfort, and anxiety/depression (all *p* = 0.000). Those with insomnia had lower self-rated health scores (73.82 ± 18.80 vs. 89.26 ± 11.95) and EQ-5D index values (0.89 ± 0.13 vs. 0.97 ± 0.85). This is in line with previous studies, highlighting the toll that insomnia exacts on an individual’s self-perception of vitality and functional capacity. Insomnia has a measurable negative impact on domains of health-related quality of life and is not limited to obvious domains, such as vitality and energy, but also affects other domains, including mental, social, and physical functioning [42]. Other studies found that insomnia is associated with work absenteeism and reduced productivity [43].

When considering the implications of the recent study, in addition to addressing insomnia, it is crucial to consider the broader framework of healthcare delivery. The quality of life of healthcare professionals is intrinsically linked to the quality of care they provide for patients. When healthcare workers experience burnout, stress, or poor mental health, it directly impacts their ability to provide compassionate and effective care. Person-centered care has been identified as essential for improving healthcare outcomes, particularly for individuals living with long-term conditions. The World Health Organization (WHO) prioritizes the re-orientation of health services around people rather than conditions or institutions. The WHO framework on integrated people-centered health services envisions services tailored to individuals’ needs and preferences, emphasizing safety, effectiveness, timeliness, affordability, and quality [44]. Person-centered practice is founded on core values such as shared autonomy, therapeutic caring, commitment to health, and respect for individuals’ abilities, preferences, and lifestyles [45]. The emphasis on equal partnerships between care providers and recipients ensures that a person’s needs are prioritized throughout all stages of care [46]. A healthy, well-supported workforce is essential for maintaining high standards of patient care. Therefore, improving the well-being of healthcare professionals should be a priority to ensure the delivery of optimal healthcare services [47,48]. To explore this connection more comprehensively, future studies should incorporate person-centered care assessment tools that evaluate both the well-being of healthcare professionals and the quality of care provided to patients. Tools such as the Person-Centered Care Assessment Tool (P-CAT) or other available tools could help assess how healthcare workers’ satisfaction, mental health, and job stress correlate with patient satisfaction and care outcomes [49,50,51].

By focusing on both the well-being of healthcare professionals and person-centered patient care, healthcare systems can foster positive environments for both providers and patients, improving health outcomes and job satisfaction. When considering the prevalence of insomnia and its related impact on healthcare professionals in our setting, an urgent implementation of support systems to prevent psychological distress among HCPs is needed. Resilience and prosocial behavior are vital in maintaining the mental health of healthcare professionals, particularly during crises [52,53]. Strengthening these traits can reduce burnout, insomnia, and stress while improving job satisfaction and patient care. A strong sense of coherence, helping individuals perceive challenges as manageable, can be a protective factor against burnout and stress [54]. To enhance this, healthcare organizations should incorporate training on coping strategies that focus on building a strong sense of coherence. This can help HCPs better handle stress by improving their perception of work challenges and fostering a sense of control over stressful situations [55]. To promote resilience, healthcare organizations should implement resilience training programs focusing on stress management, emotional regulation, and coping strategies. Creating a supportive work environment is also crucial [56]. Team-building activities, mentorship programs, and peer support groups can foster collaboration and empathy among workers, which reduces emotional strain. Work–life balance policies, such as flexible hours and mental health days, can help combat burnout. Additionally, wellness programs that promote physical and mental well-being, such as counseling services and stress-relief activities, should be prioritized. By incorporating these interventions, healthcare organizations can enhance resilience and prosocial behavior, supporting both the well-being of workers and the quality of care provided to patients [57].

Several limitations should be considered when interpreting the findings of this study. The cross-sectional design precludes any conclusions regarding causality between depression, anxiety, quality of life, burnout, and insomnia symptoms. Since data were collected using self-reported questionnaires, this may introduce recall bias and social desirability bias. The study was conducted in a single tertiary institution in Montenegro, which may limit the generalizability of the findings to healthcare professionals in other regions or healthcare settings. Factors such as pre-existing mental health conditions, workload variations between departments, and personal coping strategies were not controlled for and could have influenced the results.

## 5. Conclusions

Insomnia symptoms were prevalent in the healthcare workers in Montenegro during the pandemic and were accompanied by other mental health challenges and reduced quality of life. By addressing insomnia and associated factors, healthcare organizations can potentially improve not only the well-being of their staff but also the quality of patient care and the overall functioning of the healthcare system. This knowledge can guide the creation of targeted support programs, workplace policies, and mental health interventions in healthcare settings.

## Figures and Tables

**Table 1:** Demographic characteristics of respondents.

Characteristics	No Insomnia *n* (%)	Insomnia *n* (%)	*p*-Value
Sex			
Male	51 (35.9)	55 (35.0)	0.873
Female	91 (64.1)	102 (65.0)	
Age	36.79 ± 11.278	38.57 ± 11.567	0.180
Marital status			0.914
Single	62 (43.7)	70 (44.6)	
Married	68 (47.9)	72 (45.9)	
Divorced	12 (8.5)	15 (6.6)	
Number of children			0.487
0	73 (51.8)	72 (45.9)	
1	15 (10.6)	20 (12.7)	
2	27 (19.1)	39 (24.8)	
3	21 (14.9)	19 (12.1)	
4	5 (3.5)	7 (4.5)	
Alcohol consumption			0.007
No	122 (85.9)	115 (73.2)	
Yes	20 (14.1)	42 (26.8)	
Cigarette consumption			0.006
No	83 (58.5)	67 (42.7)	
Yes	59 (41.5)	90 (57.3)	
Consuming a sedative			0.038
No	141 (99.3)	149 (94.9)	
Yes	1 (0.7)	8 (5.1)	
Occupation			0.698
A doctor	39 (27.5)	44 (28.0)	
Nurse	79 (55.6)	81 (51.6)	
Other medical staff	24 (16.9)	32 (20.4)	
Working hours			0.107
Only in one shift	28 (19.7)	21 (13.4)	
In two shifts	29 (20.4)	21 (13.4)	
In shifts 12 h	54 (38.0)	75 (47.8)	
Morning + 24 h	31 (21.8)	40 (25.5)	
Overtime			0.321
No	47 (33.3)	44 (28.0)	
Yes	94 (66.7)	113 (72.0)	

**Table 2:** COVID-19 exposure and insomnia.

Characteristics	No Insomnia *n* (%)	Insomnia *n* (%)	*p*-Value
COVID-19 work			0.386
Not at all or continuously	111 (78.2)	129 (82.2)	
Intermintently	31 (21.8)	28 (17.8)	
COVID-19 engagement			0.097
No	29 (20.4)	27 (17.2)	
I was engaged for 1 to 3 months	26 (18.3)	18 (11.5)	
I was engaged for more than 3 months	87 (61.3)	112 (71.3)	
COVID-19 impacted my employment			0.607
I don’t agree at all	55 (39.0)	58 (37.4)	
I don’t agree	42 (29.8)	41 (26.5)	
I agree	26 (18.4)	37 (23.9)	
I completely agree	18 (12.8)	19 (12.3)	
I had increase workload and excessive engagement			0.017
I don’t agree at all	22 (15.6)	9 (5.7)	
I don’t agree	38 (27.0)	30 (19.1)	
I agree	52 (36.9)	87 (55.4)	
I completely agree	29 (20.6)	31 (19.7)	
I had adequate protective equipment			0.066
I don’t agree at all	6 (4.2)	42(1.3)	
I don’t agree	30 (21.1)	41 (26.1)	
I agree	74 (52.1)	100 (63.7)	
I completely agree	32 (22.5)	14 (8.9)	
I had enough knowledge on COVID-19			0.426
I don’t agree at all	8 (5.6)	8 (5.1)	
I don’t agree	36 (25.4)	39 (24.8)	
I agree	48 (33.8)	68 (43.3)	
I completely agree	50 (35.2)	42 (26.8)	
COVID-19 negatively impacted my finances			0.194
I don’t agree at all	31 (21.68)	28 (17.9)	
I don’t agree	48 (33.8)	42 (26.9)	
I agree	39 (27.5)	61 (39.1)	
I completely agree	24 (16.9)	25 (16.0)	
Quarantine during COVID-19			0.008
No	66 (46.5)	49 (31.4)	
Yes	76 (53.5)	107 (68.6)	
COVID-19 infection			0.001
No	90 (63.4)	69(43.9)	
Yes	52 (36.6)	88 (56.1)	

**Table 3:** EQ-5D-5L and insomnia.

Dimension	No Insomnia *n* (%)	Insomnia *n* (%)	*p*-Value
EQ-5D-5L Mobility			<0.001
I have no problems in walking about	111 (88.8)	68 (54.4)	
I have slight problems in walking about	10 (8.0)	32 (25.6)	
I have moderate problems in walking about	2 (1.6)	10 (8.0)	
I have severe problems in walking about	0 (0.0)	13 (10.4)	
I am unable to walk about	2 (1.6)	2 (1.6)	
EQ-5D-5L Self-care			<0.001
I have no problems washing or dressing myself θ	119 (95.2)	98 (78.4)	
I have slight problems washing or dressing myself θ	3 (2.4)	11 (8.8)	
I have moderate problems washing or dressing myself	1 (0.8)	16 (12.8)	
I have severe problems washing or dressing myself	2 (1.6)	0 (0.0)	
EQ-5D-5L Usual activities			<0.001
I have no problems doing my usual activities	116 (92.8)	77 (61.6)	
I have slight problems doing my usual activities	4 (3.2)	30 (24.0)	
I have moderate problems doing my usual activities	5 (4.0)	16 (12.8)	
I have severe problems doing my usual activities	0 (0.0)	2 (1.6)	
EQ-5D-5L Pain discomfort			<0.001
I have no pain or discomfort	98 (78.4)	49 (39.2)	
I have slight pain or discomfort	20 (16.0)	40 (32.0)	
I have moderate pain or discomfort	6 (4.8)	27 (21.6)	
I have severe pain or discomfort	1 (0.8)	9 (7.2)	
EQ-5D-5L Anxiety and depression			<0.001
I am not anxious or depressed	116 (92.8)	62 (49.6)	
I am slightly anxious or depressed	7 (5.6)	43 (34.4)	
I am moderately anxious or depressed	2 (1.6)	15 (12.0)	
I am severely anxious or depressed	0 (0.0)	5 (4.0)	
EQ-5D-5L Health condition	89.26 ± 11.949	73.82 ± 18.799	<0.001
EQ-5D-5L Index	0.97 ± 0.85	0.89 ± 0.13	<0.001

**Table 4:** DASS mental assessment and insomnia.

Characteristics	No Insomnia *n* (%)	Insomnia *n* (%)	*p*-Value
ASS21 stress level			<0.001
Normal	115 (81.0)	97 (61.8)	
Mild	13 (9.2)	21 (13.4)	
Moderate	10 (7.0)	26 (16.6)	
Severe	3 (2.1)	11 (7.0)	
Extremely severe	1 (0.7)	2 (1.3)	
ASS21 anxiety level			<0.001
Normal	103(72.5)	78 (49.7)	
Mild	6 (4.2)	10 (6.4)	
Moderate	21 (14.8)	27 (17.2)	
Severe	4 (2.8)	20 (12.7)	
Extremely severe	8 (5.6)	22 (14.0)	
ASS21 degree of depression			<0.001
Normal	117 (82.4)	97 (61.8)	
Mild	14 (9.9)	20 (12.7)	
Moderate	10 (7.0)	29 (18.5)	
Severe	1(0.7)	9 (5.7)	
Extremely severe	0 (0.0)	2 (1.3)	

## Data Availability

Data are available from the main author based on individual requests.

## Supplementary Materials

The following supporting information can be downloaded at https://www.mdpi.com/article/10.3390/jcm14103374/s1: File S1: The data collection instruments used.
