# Supplementary material for: Association Between Depression, Anxiety, Quality of Life and Burnout Syndrome with Symptoms of Insomnia in Healthcare Professionals in Montenegro During the COVID-19 Pandemic
Source: J Clin Med. 2025 May 12;14(10):3374. doi: 10.3390/jcm14103374 (PMC12112652; doi:10.3390/jcm14103374)
Supplement: Supplementary file 1 [file jcm-14-03374-s001.zip › jcm-3596249-supplementary.pdf]

# 1. Prilozi

Prilog 1. Opšti upitnik za prikupljanje socio-demografskih podataka

**SOCIO-DEMOGRAFSKI PODACI**

**Indentifikacioni broj 001**

1. **Zaokružite Vaš pol? M Ž**

2. **Koliko imate godina?**

   **---------------------------------------------**

3. **Koji je Vaš bračni status?**

- **Neoženjen/neudata**
- **Oženjen/udata**
- **Razveden/a**
- **Udovac/ica**

4. **Da li imate djece(ako imate upisati broj djece)**

5. **Konzumiram?**

- **Alkohol**
- **Cigarete**
- **Sedative**

6. **Zanimanje?**

- **Stručni stepen ljekara**
- **Ljekar bez specijalizacije**
- **Ljekar na specijalizaci...............................(navesti spacijalizaciju)**
- **Ljekar specijalista…..................................(navesti)**
- **Medicinska sestra/tehničar**
- **Ostalo …………………….**

7. **Stručno/naučno zvanje ljekara?**

- **Prim.Dr**
- **Mr sci med**
- **Dr. sci. med.**
- **Doc. Dr.**
- **Prf. Dr.**

8. **Stručno zvanje medicinskih sestara?**

- **SSS**
- **VMS**
- **VSS**
- **Strukovana medicinska sestra**
- **Magistar zdravstvene njege**
- **Doktorant zdravstvene njege**

9. **Naziv organizacione jedinice………………………………………………………**

10. **Za vrijeme kovid pandemije u kojoj organizacionoj jedinici ste bili angažovani :**

**------------------------------------------------------------------------------------------**

11. **Dužina ukupnog radnog staža ----------- godina.**

12. **Radno vrijeme?**

- **Samo u jutarnjoj smjeni**
- **Samo u popodnevnoj smjeni**
- **Samo u noćnoj smjeni**
- **U dvije smjene (jutarnja, popodnevna)**
- **U smjene od 12h (dnevna/noćna)**
- **Jutarnja smjena+dežurstvo (24 h)**
- **Drugo (navesti što?)………………….**

13. **Da li radite dopunski ili prekovremeno? DA NE**

14. **Radim:**

- **U timu**
- **Samostalno**

15. **Jeste li zaposleni na neodređeno vrijeme?   DA   NE**

16. **Tokom COVIDA 19 epidemije sam opterećen/a pretjeranima agažovanjem na poslu kao i povećanim brojem radnih sati?**

**Uopšte se ne slažem   ne slažem se   slažem se   u potpunosti se slažem**

17. **Tokom COVID 19 pandemije sam se osjećalo/a bezbjednim i adekvatno opremljeno na svom random mjestu.**

**Uopšte se ne slažem   ne slažem se   slažem se   u potpunosti se slažem**

18. **Tokom COVID 19 pandemije sam se osjećala da raspolažem sa dovoljno znanja i informacija da odgovorim aktuelnim izazovima.**

**Uopšte se ne slažem   ne slažem se   slažem se   u potpunosti se slažem**

19. **Molim Vas da upišete koliko ste vremena bili angažovani u radu sa COVID pozitivnim pacijentima za vrijeme COVID pandemije.**

- **Nisam angažovana za rad oko COVID pacijenata:**
- **Angažovana sam do mjesec dana;**
- **Angažovana sam od 1 do 3 mjeseca;**
- **Angažovana sam izmedju 3 i 6 mjeseci;**
- **Angažovana sam više od 6 mjeseci.**
- **Angažovana sam više od 12 mjeseci.**

**20. Da li je COVID 19 Pandemija uticala na Vaše zaposlenje.**

**Uopšte se ne slažem ne slažem se slažem se u potpunosti se slažem**

**21. Da li je COVID 19 Pandemija uticala na Vas finasijski.**

**Uopšte se ne slažem ne slažem se slažem se u potpunosti se slažem**

**22. Da li ste bili u karantinu**

**A) Da;**

**B) Ne.**

**Ako je odgovor DA**

**A) Zbog infekcije COVIDOM 19;**

**B) Kao kontakt.**

**23. Slijedio/la sam preporuku IJZCG za vakciju protiv COVIDA 19.**

**Uopšte se ne slažem ne slažem se slažem se u potpunosti se slažem**

Prilog 2. Upitnik za procenu sindroma sagorijevanja na poslu - Maslach Burnout Inventory

For use by Dragana Backovic only. Received from Mind Garden, Inc. on January 27, 2020

**Permission for Dragana Backovic to reproduce 1 copy**
**within one year of January 27, 2020**

# Maslach Burnout Inventory

## Instruments and Scoring Guides

## *English:* MBI-GS, MBI-HSS, MBI-ES forms

## *Serbian:* MBI-GS, MBI-HSS, MBI-ES forms

Christina Maslach
Susan E. Jackson
Michael P. Leiter
Wilmar B. Schaufeli
Richard L. Schwab

**Published by Mind Garden**

info@mindgarden.com
www.mindgarden.com

## Important Note to Licensee

If you have purchased a license to reproduce or administer a fixed number of copies of an existing Mind Garden instrument, manual, or workbook, you agree that it is your legal responsibility to compensate the copyright holder of this work — via payment to Mind Garden — for reproduction or administration in any medium. **Reproduction includes all forms of physical or electronic administration including online survey, handheld survey devices, etc.**

The copyright holder has agreed to grant a license to reproduce the specified number of copies of this document or instrument **within one year from the date of purchase.**

**You agree that you or a person in your organization will be assigned to track the number of reproductions or administrations and will be responsible for compensating Mind Garden for any reproductions or administrations in excess of the number purchased.**

*This instrument is covered by U.S. and international copyright laws as well as various state and federal laws regarding data protection. Any use of this instrument, in whole or in part, is subject to such laws and is expressly prohibited by the copyright holder. If you would like to request permission to use or reproduce the instrument, in whole or in part, contact Mind Garden, Inc.*

**MBI-General Survey:** Copyright ©1996 Wilmar B. Schaufeli, Michael P. Leiter, Christina Maslach & Susan E. Jackson.
**MBI-Human Services Survey:** Copyright ©1981 Christina Maslach & Susan E. Jackson.
**MBI-Educators Survey:** Copyright ©1986 Christina Maslach, Susan E. Jackson & Richard L. Schwab.
All rights reserved in all media. Published by Mind Garden, Inc., www.mindgarden.com

For use by  Dragana Backovic only. Received from Mind Garden, Inc. on January 27, 2020

## Translation quality

Mind Garden distributed translations are of varying quality. Many are translated by researchers. Some translations are translated and then back-translated to check the quality, while others have not been back-translated. We typically do not know the dialect of the translation. We also do not have validation data on many translations. Some translations do not include all of the items that are on the English form; sometimes a scale or scales are missing on the translation. You will receive what we have with no warranty or assurance of quality or dialect. Basically, we try to provide you with what is available to facilitate your work.

**MBI-General Survey:** Copyright ©1996 Wilmar B. Schaufeli, Michael P. Leiter, Christina Maslach & Susan E. Jackson.
**MBI-Human Services Survey:** Copyright ©1981 Christina Maslach & Susan E. Jackson.
**MBI-Educators Survey:** Copyright ©1986 Christina Maslach, Susan E. Jackson & Richard L. Schwab.
All rights reserved in all media.  Published by Mind Garden, Inc., www.mindgarden.com

For use by Dragana Backovic only. Received from Mind Garden, Inc. on January 27, 2020

# MBI-General Survey

Wilmar B. Schaufeli, Michael P. Leiter, Christina Maslach & Susan E. Jackson

*The purpose of this survey is to discover how staff members view their job, and their reactions to their work.*

**Instructions:** On the following page are 16 statements of job-related feelings. Please read each statement carefully and decide if you ever feel this way about *your* job. If you have *never* had this feeling, write the number "0" (zero) in the space before the statement. If you have had this feeling, indicate *how often* you feel it by writing the number (from 1 to 6) that best describes how frequently you feel that way. An example is shown below.

**Example:**

| **How often:** | **0** | **1** | **2** | **3** | **4** | **5** | **6** |
|---|---|---|---|---|---|---|---|
| | Never | A few times a year or less | Once a month or less | A few times a month | Once a week | A few times a week | Every day |

| **How Often 0-6** | **Statement:** |
|---|---|
| 1. _________ | I feel depressed at work. |

If you never feel depressed at work, you would write the number "0" (zero) under the heading "How Often." If you rarely feel depressed at work (a few times a year or less), you would write the number "1." If your feelings of depression are fairly frequent (a few times a week but not daily), you would write the number "5."

**MBI-General Survey:** Copyright ©1996 Wilmar B. Schaufeli, Michael P. Leiter, Christina Maslach & Susan E. Jackson.
**MBI-Human Services Survey:** Copyright ©1981 Christina Maslach & Susan E. Jackson.
**MBI-Educators Survey:** Copyright ©1986 Christina Maslach, Susan E. Jackson & Richard L. Schwab.
All rights reserved in all media. Published by Mind Garden, Inc., www.mindgarden.com

For use by Dragana Backovic only. Received from Mind Garden, Inc. on January 27, 2020

# MBI-General Survey

| **How often:** | **0** | **1** | **2** | **3** | **4** | **5** | **6** |
| --- | --- | --- | --- | --- | --- | --- | --- |
| | Never | A few times a year or less | Once a month or less | A few times a month | Once a week | A few times a week | Every day |

| **How Often 0-6** | **Statements:** |
| --- | --- |
| 1. ________ | I feel emotionally drained from my work. |
| 2. ________ | I feel used up at the end of the workday. |
| 3. ________ | I feel tired when I get up in the morning and have to face another day on the job. |
| 4. ________ | Working all day is really a strain for me. |
| 5. ________ | I can effectively solve the problems that arise in my work. |
| 6. ________ | I feel burned out from my work. |
| 7. ________ | I feel I am making an effective contribution to what this organization does. |
| 8. ________ | I've become less interested in my work since I started this job. |
| 9. ________ | I have become less enthusiastic about my work. |
| 10. ________ | In my opinion, I am good at my job. |
| 11. ________ | I feel exhilarated when I accomplish something at work. |
| 12. ________ | I have accomplished many worthwhile things in this job. |
| 13. ________ | I just want to do my job and not be bothered. |
| 14. ________ | I have become more cynical about whether my work contributes anything. |
| 15. ________ | I doubt the significance of my work. |
| 16. ________ | At my work, I feel confident that I am effective at getting things done. |

(Administrative use only)

**EX:** _______ cat:_______ **CY:** _______ cat:_______ **PE:** _______ cat:_______

**MBI-General Survey:** Copyright ©1996 Wilmar B. Schaufeli, Michael P. Leiter, Christina Maslach & Susan E. Jackson.
**MBI-Human Services Survey:** Copyright ©1981 Christina Maslach & Susan E. Jackson.
**MBI-Educators Survey:** Copyright ©1986 Christina Maslach, Susan E. Jackson & Richard L. Schwab.
All rights reserved in all media. Published by Mind Garden, Inc., www.mindgarden.com

For use by Dragana Backovic only. Received from Mind Garden, Inc. on January 27, 2020

# MBI–General Survey Scoring Key

## Professional Efficacy (PE) Subscale

**Directions:** Line up the item numbers on this key with the same numbers on the survey form. Looking at the unshaded items only, add the scores in the "How Often" column and enter the total in the "PE" space at the bottom of the survey form.

| How Often 0-6 | |
|---|---|
| 1. | ________ |
| 2. | ________ |
| 3. | ________ |
| 4. | ________ |
| 5. | ________ |
| 6. | ________ |
| 7. | ________ |
| 8. | ________ |
| 9. | ________ |
| 10. | ________ |
| 11. | ________ |
| 12. | ________ |
| 13. | ________ |
| 14. | ________ |
| 15. | ________ |
| 16. | ________ |

| Categorization: Professional Efficacy* | |
|---|---|
| | Frequency |
| High | 30 or over |
| Moderate | 24-29 |
| Low | 0-23 |

*Interpreted in the opposite direction from EX and CY.

Note to Researchers: Research reports using the MBI—General Survey usually report the average rating rather than the total. To determine the average rating for each subscale, divide the total by the number of items responded to. The Professional Efficacy scale contains 6 items.

**MBI-General Survey:** Copyright ©1996 Wilmar B. Schaufeli, Michael P. Leiter, Christina Maslach & Susan E. Jackson.
**MBI-Human Services Survey:** Copyright ©1981 Christina Maslach & Susan E. Jackson.
**MBI-Educators Survey:** Copyright ©1986 Christina Maslach, Susan E. Jackson & Richard L. Schwab.
All rights reserved in all media. Published by Mind Garden, Inc., www.mindgarden.com

For use by Dragana Backovic only. Received from Mind Garden, Inc. on January 27, 2020

# MBI–General Survey Scoring Key

## Exhaustion (EX) Subscale

**Directions:** Line up the item numbers on this key with the same numbers on the survey form. Looking at the unshaded items only, add the scores in the "How Often" column and enter the total in the "EX" space at the bottom of the survey form.

| How Often 0-6 |
|---|
| 1. ________ |
| 2. ________ |
| 3. ________ |
| 4. ________ |
| 5. ________ |
| 6. ________ |
| 7. ________ |
| 8. ________ |
| 9. ________ |
| 10. ________ |
| 11. ________ |
| 12. ________ |
| 13. ________ |
| 14. ________ |
| 15. ________ |
| 16. ________ |

| Categorization: Exhaustion | |
|---|---|
| | Frequency |
| High | 16 or over |
| Moderate | 11-15 |
| Low | 0-10 |

Note to Researchers: Research reports using the MBI—General Survey usually report the average rating rather than the total. To determine the average rating for each subscale, divide the total by the number of items responded to. The Exhaustion scale contains 5 items.

**MBI-General Survey**: Copyright ©1996 Wilmar B. Schaufeli, Michael P. Leiter, Christina Maslach & Susan E. Jackson.
**MBI-Human Services Survey**: Copyright ©1981 Christina Maslach & Susan E. Jackson.
**MBI-Educators Survey**: Copyright ©1986 Christina Maslach, Susan E. Jackson & Richard L. Schwab.
All rights reserved in all media. Published by Mind Garden, Inc., www.mindgarden.com

For use by Dragana Backovic only. Received from Mind Garden, Inc. on January 27, 2020

# MBI–General Survey Scoring Key

## Cynicism (CY) Subscale

**Directions:** Line up the item numbers on this key with the same numbers on the survey form. Looking at the unshaded items only, add the scores in the "How Often" column and enter the total in the "CY" space at the bottom of the survey form.

| How Often 0-6 |
|---|
| 1. ________ |
| 2. ________ |
| 3. ________ |
| 4. ________ |
| 5. ________ |
| 6. ________ |
| 7. ________ |
| 8. ________ |
| 9. ________ |
| 10. ________ |
| 11. ________ |
| 12. ________ |
| 13. ________ |
| 14. ________ |
| 15. ________ |
| 16. ________ |

| Categorization: Cynicism | |
|---|---|
| | Frequency |
| High | 11 or over |
| Moderate | 6-10 |
| Low | 0-5 |

Note to Researchers: Research reports using the MBI—General Survey usually report the average rating rather than the total. To determine the average rating for each subscale, divide the total by the number of items responded to. The Cynicism scale contains 5 items.

**MBI-General Survey:** Copyright ©1996 Wilmar B. Schaufeli, Michael P. Leiter, Christina Maslach & Susan E. Jackson.
**MBI-Human Services Survey:** Copyright ©1981 Christina Maslach & Susan E. Jackson.
**MBI-Educators Survey:** Copyright ©1986 Christina Maslach, Susan E. Jackson & Richard L. Schwab.
All rights reserved in all media. Published by Mind Garden, Inc., www.mindgarden.com

For use by Dragana Backovic only. Received from Mind Garden, Inc. on January 27, 2020

# MBI-Human Services Survey

Christina Maslach & Susan E. Jackson

*The purpose of this survey is to discover how various persons in the human services, or helping professionals view their job and the people with whom they work closely.*

Because persons in a wide variety of occupations will answer this survey, it uses the term *recipients* to refer to the people for whom you provide your service, care, treatment, or instruction. When answering this survey please think of these people as recipients of the service you provide, even though you may use another term in your work.

**Instructions:** On the following pages are 22 statements of job-related feelings. Please read each statement carefully and decide if you ever feel this way about *your* job. If you have *never* had this feeling, write the number "0" (zero) in the space before the statement. If you have had this feeling, indicate *how often* you feel it by writing the number (from 1 to 6) that best describes how frequently you feel that way. An example is shown below.

**Example:**

| **How often:** | **0** | **1** | **2** | **3** | **4** | **5** | **6** |
|---|---|---|---|---|---|---|---|
| | Never | A few times a year or less | Once a month or less | A few times a month | Once a week | A few times a week | Every day |

| **How Often 0-6** | **Statement:** |
|---|---|
| 1. __________ | I feel depressed at work. |

If you never feel depressed at work, you would write the number "0" (zero) under the heading "How Often." If you rarely feel depressed at work (a few times a year or less), you would write the number "1." If your feelings of depression are fairly frequent (a few times a week but not daily), you would write the number "5."

**MBI-General Survey:** Copyright ©1996 Wilmar B. Schaufeli, Michael P. Leiter, Christina Maslach & Susan E. Jackson.
**MBI-Human Services Survey:** Copyright ©1981 Christina Maslach & Susan E. Jackson.
**MBI-Educators Survey:** Copyright ©1986 Christina Maslach, Susan E. Jackson & Richard L. Schwab.
All rights reserved in all media. Published by Mind Garden, Inc., www.mindgarden.com

For use by Dragana Backovic only. Received from Mind Garden, Inc. on January 27, 2020

# MBI-Human Services Survey

| How often: | 0 | 1 | 2 | 3 | 4 | 5 | 6 |
|---|---|---|---|---|---|---|---|
| | Never | A few times a year or less | Once a month or less | A few times a month | Once a week | A few times a week | Every day |

| How Often 0-6 | Statements: |
|---|---|
| 1. ________ | I feel emotionally drained from my work. |
| 2. ________ | I feel used up at the end of the workday. |
| 3. ________ | I feel fatigued when I get up in the morning and have to face another day on the job. |
| 4. ________ | I can easily understand how my recipients feel about things. |
| 5. ________ | I feel I treat some recipients as if they were impersonal objects. |
| 6. ________ | Working with people all day is really a strain for me. |
| 7. ________ | I deal very effectively with the problems of my recipients. |
| 8. ________ | I feel burned out from my work. |
| 9. ________ | I feel I'm positively influencing other people's lives through my work. |
| 10. ________ | I've become more callous toward people since I took this job. |
| 11. ________ | I worry that this job is hardening me emotionally. |
| 12. ________ | I feel very energetic. |
| 13. ________ | I feel frustrated by my job. |
| 14. ________ | I feel I'm working too hard on my job. |
| 15. ________ | I don't really care what happens to some recipients. |
| 16. ________ | Working with people directly puts too much stress on me. |
| 17. ________ | I can easily create a relaxed atmosphere with my recipients. |
| 18. ________ | I feel exhilarated after working closely with my recipients. |
| 19. ________ | I have accomplished many worthwhile things in this job. |
| 20. ________ | I feel like I'm at the end of my rope. |
| 21. ________ | In my work, I deal with emotional problems very calmly. |
| 22. ________ | I feel recipients blame me for some of their problems. |

(Administrative use only)

**EE:** _______ cat:_______ **DP:** _______ cat:_______ **PA:** _______ cat:_______

**MBI-General Survey:** Copyright ©1996 Wilmar B. Schaufeli, Michael P. Leiter, Christina Maslach & Susan E. Jackson.
**MBI-Human Services Survey:** Copyright ©1981 Christina Maslach & Susan E. Jackson.
**MBI-Educators Survey:** Copyright ©1986 Christina Maslach, Susan E. Jackson & Richard L. Schwab.
All rights reserved in all media. Published by Mind Garden, Inc., www.mindgarden.com

For use by Dragana Backovic only. Received from Mind Garden, Inc. on January 27, 2020

# MBI-Educators Survey

Christina Maslach, Susan E. Jackson & Richard L. Schwab

*The purpose of this survey is to discover how educators view their job and the people with whom they work closely.*

**Instructions:** On the following pages are 22 statements of job-related feelings. Please read each statement carefully and decide if you ever feel this way about *your* job. If you have *never* had this feeling, write the number "0" (zero) in the space before the statement. If you have had this feeling, indicate *how often* you feel it by writing the number (from 1 to 6) that best describes how frequently you feel that way. An example is shown below.

| How often: | 0 | 1 | 2 | 3 | 4 | 5 | 6 |
|---|---|---|---|---|---|---|---|
| | Never | A few times a year or less | Once a month or less | A few times a month | Once a week | A few times a week | Every day |

**Example:**

| How Often 0-6 | Statement: |
|---|---|
| 1. ________ | I feel depressed at work. |

If you never feel depressed at work, you would write the number "0" (zero) under the heading "How Often." If you rarely feel depressed at work (a few times a year or less), you would write the number "1." If your feelings of depression are fairly frequent (a few times a week but not daily), you would write the number "5."

**MBI-General Survey:** Copyright ©1996 Wilmar B. Schaufeli, Michael P. Leiter, Christina Maslach & Susan E. Jackson.
**MBI-Human Services Survey:** Copyright ©1981 Christina Maslach & Susan E. Jackson.
**MBI-Educators Survey:** Copyright ©1986 Christina Maslach, Susan E. Jackson & Richard L. Schwab.
All rights reserved in all media. Published by Mind Garden, Inc., www.mindgarden.com

For use by Dragana Backovic only. Received from Mind Garden, Inc. on January 27, 2020

# MBI-Educators Survey

| How often: | 0 | 1 | 2 | 3 | 4 | 5 | 6 |
|---|---|---|---|---|---|---|---|
| | Never | A few times a year or less | Once a month or less | A few times a month | Once a week | A few times a week | Every day |

| How Often 0-6 | Statements: |
|---|---|
| 1. ________ | I feel emotionally drained from my work. |
| 2. ________ | I feel used up at the end of the workday. |
| 3. ________ | I feel fatigued when I get up in the morning and have to face another day on the job. |
| 4. ________ | I can easily understand how my students feel about things. |
| 5. ________ | I feel I treat some students as if they were impersonal objects. |
| 6. ________ | Working with people all day is really a strain for me. |
| 7. ________ | I deal very effectively with the problems of my students. |
| 8. ________ | I feel burned out from my work. |
| 9. ________ | I feel I'm positively influencing other people's lives through my work. |
| 10. ________ | I've become more callous toward people since I took this job. |
| 11. ________ | I worry that this job is hardening me emotionally. |
| 12. ________ | I feel very energetic. |
| 13. ________ | I feel frustrated by my job. |
| 14. ________ | I feel I'm working too hard on my job. |
| 15. ________ | I don't really care what happens to some students. |
| 16. ________ | Working with people directly puts too much stress on me. |
| 17. ________ | I can easily create a relaxed atmosphere with my students. |
| 18. ________ | I feel exhilarated after working closely with my students. |
| 19. ________ | I have accomplished many worthwhile things in this job. |
| 20. ________ | I feel like I'm at the end of my rope. |
| 21. ________ | In my work, I deal with emotional problems very calmly. |
| 22. ________ | I feel students blame me for some of their problems. |

(Administrative use only)

**EE:** ________ cat: ________ **DP:** ________ cat: ________ **PA:** ________ cat: ________

**MBI-General Survey:** Copyright ©1996 Wilmar B. Schaufeli, Michael P. Leiter, Christina Maslach & Susan E. Jackson.
**MBI-Human Services Survey:** Copyright ©1981 Christina Maslach & Susan E. Jackson.
**MBI-Educators Survey:** Copyright ©1986 Christina Maslach, Susan E. Jackson & Richard L. Schwab.
All rights reserved in all media. Published by Mind Garden, Inc., www.mindgarden.com

For use by Dragana Backovic only. Received from Mind Garden, Inc. on January 27, 2020

# MBI–Human Services and Educators Scoring Key
## Emotional Exhaustion (EE) Subscale

**Directions:** Line up the item numbers on this key with the same numbers on the survey form. Looking at the unshaded items only, add the scores in the "How Often" column and enter the total in the "EE" space at the bottom of the survey form.

| How Often 0-6 |
|---|
| 1. ________ |
| 2. ________ |
| 3. ________ |
| 4. ________ |
| 5. ________ |
| 6. ________ |
| 7. ________ |
| 8. ________ |
| 9. ________ |
| 10. ________ |
| 11. ________ |
| 12. ________ |
| 13. ________ |
| 14. ________ |
| 15. ________ |
| 16. ________ |
| 17. ________ |
| 18. ________ |
| 19. ________ |
| 20. ________ |
| 21. ________ |
| 22. ________ |

| Categorization: Emotional Exhaustion, Human Services & Educators Forms | |
|---|---|
| | Frequency |
| High | 27 or over |
| Moderate | 17-26 |
| Low | 0-16 |

Note to Researchers: Research reports using the MBI—Human Services & Educators Forms usually report the average rating rather than the total. To determine the average rating for each subscale, divide the total by the number of items responded to. The Emotional Exhaustion scale contains 9 items.

**MBI-General Survey:** Copyright ©1996 Wilmar B. Schaufeli, Michael P. Leiter, Christina Maslach & Susan E. Jackson.
**MBI-Human Services Survey:** Copyright ©1981 Christina Maslach & Susan E. Jackson.
**MBI-Educators Survey:** Copyright ©1986 Christina Maslach, Susan E. Jackson & Richard L. Schwab.
All rights reserved in all media. Published by Mind Garden, Inc., www.mindgarden.com

For use by Dragana Backovic only. Received from Mind Garden, Inc. on January 27, 2020

# MBI–Human Services and Educators Scoring Key

## Depersonalization (DP) Subscale

**Directions:** Line up the item numbers on this key with the same numbers on the survey form. Looking at the unshaded items only, add the scores in the "How Often" column and enter the total in the "DP" space at the bottom of the survey form.

| How Often 0-6 |
|---|
| 1. ________ |
| 2. ________ |
| 3. ________ |
| 4. ________ |
| 5. ________ |
| 6. ________ |
| 7. ________ |
| 8. ________ |
| 9. ________ |
| 10. ________ |
| 11. ________ |
| 12. ________ |
| 13. ________ |
| 14. ________ |
| 15. ________ |
| 16. ________ |
| 17. ________ |
| 18. ________ |
| 19. ________ |
| 20. ________ |
| 21. ________ |
| 22. ________ |

| Categorization: Depersonalization, Human Services Form | | Categorization: Depersonalization, Educators Form | |
|---|---|---|---|
| | Frequency | | Frequency |
| High | 13 or over | High | 14 or over |
| Moderate | 7-12 | Moderate | 9-13 |
| Low | 0-6 | Low | 0-8 |

Note to Researchers: Research reports using the MBI—Human Services & Educators Forms usually report the average rating rather than the total. To determine the average rating for each subscale, divide the total by the number of items responded to. The Depersonalization scale contains 5 items.

**MBI-General Survey:** Copyright ©1996 Wilmar B. Schaufeli, Michael P. Leiter, Christina Maslach & Susan E. Jackson.
**MBI-Human Services Survey:** Copyright ©1981 Christina Maslach & Susan E. Jackson.
**MBI-Educators Survey:** Copyright ©1986 Christina Maslach, Susan E. Jackson & Richard L. Schwab.
All rights reserved in all media. Published by Mind Garden, Inc., www.mindgarden.com

For use by Dragana Backovic only. Received from Mind Garden, Inc. on January 27, 2020

# MBI–Human Services and Educators Scoring Key
## Personal Accomplishment (PA) Subscale

**Directions:** Line up the item numbers on this key with the same numbers on the survey form. Looking at the unshaded items only, add the scores in the "How Often" column and enter the total in the "PA" space at the bottom of the survey form.

| How Often 0-6 |
|---|
| 1. ________ |
| 2. ________ |
| 3. ________ |
| 4. ________ |
| 5. ________ |
| 6. ________ |
| 7. ________ |
| 8. ________ |
| 9. ________ |
| 10. ________ |
| 11. ________ |
| 12. ________ |
| 13. ________ |
| 14. ________ |
| 15. ________ |
| 16. ________ |
| 17. ________ |
| 18. ________ |
| 19. ________ |
| 20. ________ |
| 21. ________ |
| 22. ________ |

| Categorization: Personal Accomplishment,* Human Services Form | | Categorization: Personal Accomplishment,* Educators Form | |
|---|---|---|---|
| | Frequency | | Frequency |
| High | 39 or over | High | 37 or over |
| Moderate | 32-38 | Moderate | 31-36 |
| Low | 0 - 31 | Low | 0 - 30 |

*Interpreted in opposite direction from EE and DP. Note to Researchers: Research reports using the MBI—Human Services & Educators Forms usually report the average rating rather than the total. To determine the average rating for each subscale, divide the total by the number of items responded to. The Personal Accomplishments scale contains 8 items.

**MBI-General Survey:** Copyright ©1996 Wilmar B. Schaufeli, Michael P. Leiter, Christina Maslach & Susan E. Jackson.
**MBI-Human Services Survey:** Copyright ©1981 Christina Maslach & Susan E. Jackson.
**MBI-Educators Survey:** Copyright ©1986 Christina Maslach, Susan E. Jackson & Richard L. Schwab.
All rights reserved in all media. Published by Mind Garden, Inc., www.mindgarden.com

For use by Dragana Backovic only. Received from Mind Garden, Inc. on January 27, 2020

# MBI-GS

| 0 | 1 | 2 | 3 | 4 | 5 | 6 |
|---|---|---|---|---|---|---|
| nikad | nekoliko puta godišnje | jednom mesečno ili manje | nekoliko puta mesečno | jednom nedeljno | nekoliko puta nedeljno | svakog dana |

1. Osećam se emocionalno iscrpljeno zbog svog posla
2. Osećam se potrošeno na kraju radnog dana
3. Umoran sam već kad se probudim i moram da se suočim sa još jednim radnim danom
4. Svakodnevni rad je zaista naporan za mene
5. Efikasno rešavam probleme koji nastaju u mom radu
6. Osećam se "sagorelo" zbog svog posla
7. Osećam da pružam značajan doprinos organizaciji u kojoj radim
8. Manje se interesujem za posao od kada sam počeo da se bavim istim
9. Postao sam manje oduševljen svojim poslom
10. Po mom mišljenju, dobro obavljam svoj posao
11. Ushićen sam kad na poslu nešto postignem
12. Dobio sam mnogo toga vrednog od ovog posla
13. Samo želim da radim svoj posao i da me ne uznemiravaju ni oko čega drugog
14. Manje mi je bitno, nego pre, da li moj rad nečemu doprinosi
15. Sumnjam u značaj svog rada
16. Siguran sam da sam efikasan u izvršavanju svog posla

TA-221 – MBI – General Survey - all 16 items - Serbian

**MBI-General Survey**: Copyright ©1996 Wilmar B. Schaufeli, Michael P. Leiter, Christina Maslach & Susan E. Jackson.
**MBI-Human Services Survey**: Copyright ©1981 Christina Maslach & Susan E. Jackson.
**MBI-Educators Survey**: Copyright ©1986 Christina Maslach, Susan E. Jackson & Richard L. Schwab.
All rights reserved in all media. Published by Mind Garden, Inc., www.mindgarden.com

For use by Dragana Backovic only. Received from Mind Garden, Inc. on January 27, 2020

# MBI-HSS

| 0 | 1 | 2 | 3 | 4 | 5 | 6 |
|---|---|---|---|---|---|---|
| nikad | nekoliko puta godišnje | jednom mesečno ili manje | nekoliko puta mesečno | jednom nedeljno | nekoliko puta nedeljno | svakog dana |

1. Osećam se emocionalno iscrpljeno zbog svog posla
2. Osećam se potrošeno na kraju radnog dana
3. Umoran sam već kad se probudim i moram da se suočim sa još jednim radnim danom
4. S lakoćom razumem kako se moji pacijenti osećaju
5. Nekada imam bezličan, mehanički odnos prema pacijentima
6. Svakodnevni rad sa ljudima je zaista naporan za mene
7. Veoma efikasno rešavam probleme mojih pacijenata
8. Osećam se "sagorelo" zbog svog posla
9. Osećam da, svojim radom, pozitivno utičem na živote drugih ljudi
10. Postao sam bezosećajan ( "krut") prema ljudima od kada se bavim ovim poslom
11. Mislim da me ovaj posao emocionalno zatupljuje
12. Osećam da sam pun energije
13. Isfrustriran sam svojim poslom
14. Osećam da mi je na poslu suviše naporno
15. Nije mi stalo do pojedinih pacijenata
16. Direktan kontakt s ljudima na poslu je suviše stresan za mene
17. S lakoćom stvaram opuštenu atmosferu pri radu s pacijentima
18. Raspoložen sam nakon rada sa mojim pacijentima
19. Dobio sam mnogo toga vrednog od ovog posla
20. Osećam da sam na granici izdržljivosti
21. Mogu mirno da se nosim s emocionalnim problemima na svom poslu
22. Osećam da me pacijenti krive zbog svojih problema

TA-222 – MBI – Human Services Survey - all 22 items - Serbian

**MBI-General Survey:** Copyright ©1996 Wilmar B. Schaufeli, Michael P. Leiter, Christina Maslach & Susan E. Jackson.
**MBI-Human Services Survey:** Copyright ©1981 Christina Maslach & Susan E. Jackson.
**MBI-Educators Survey:** Copyright ©1986 Christina Maslach, Susan E. Jackson & Richard L. Schwab.
All rights reserved in all media. Published by Mind Garden, Inc., www.mindgarden.com

For use by Dragana Backovic only. Received from Mind Garden, Inc. on January 27, 2020

**MBI Upitnik za merenje burnouta Kristine Maslač**

Koliko često: 0 nikad
1 nekoliko puta godišnje ili manje
2 jednom mesečno ili manje
3 nekoliko puta mesečno
4 jednom sedmično
5 nekoliko puta sedmično
6 svakodnevno

1. Osećam se emotivno isceđen/a od mog posla
2. Osećam se potrošeno na kraju dana
3. Osećam se umorno izjutra kad ustanem i moram da se suočim sa još jednim radnim danom
4. Sa lakoćom mogu da razumem kako se moji klijenti osećaju povodom raznih stvari
5. Osećam da pojedine klijente tretiram kao da su bezlični objekti
6. Raditi sa ljudima po ceo dan za mene je veliki napor
7. Vrlo efikasno se nosim sa problemima svojih klijenata
8. Osećan se sagoreo/la od posla
9. Osećam da pozitivno utičem na živote drugih ljudi kroz svoj posao
10. Postao/la sam bezosećajniji/a prema ljudima od kako radim ovaj posao
11. Brinem se da me ovaj posao čini emotivno grubljim/om
12. Osećam se punim/om snage
13. Osećam se isfrustriranim/om svojim poslom
14. Mislim da prenaporno radim na poslu
15. Nije me stvarno briga šta će se desiti sa pojedinim klijentima
16. Neposredni rad sa ljudima je previše stresan za mene
17. Lako mogu da stvorim opuštenu atmosferu sa klijentima
18. Raspoložen/a sam i vedriji/a nakon bliskog kontakta sa klijentima
19. Postigao/la sam mnogo vrednih stvari radeći ovaj posao
20. Osećam se kao da sam na izmaku snaga
21. Na poslu veoma smireno izlazim na kraj sa emocionalnim problemima
22. Osećam da me klijenti okrivljuju za neke od svojih problema

Emocionalna iscrpljenost __ Stepen
Depersona,izacija __ Stepen
Lična profesionalna ostvarenost __ Stepen

MBI Istraživanje u humanističkim službama

*Svrha ovog istraživanja je ispitivanje kako različite ososbe zaposlene u humanističkim službama , ili pomažućim službama doživljavaju svoj posao i ljude sa kojima rade*

**TA-213 MBI-HSS - Serbian**

**MBI-General Survey:** Copyright ©1996 Wilmar B. Schaufeli, Michael P. Leiter, Christina Maslach & Susan E. Jackson.
**MBI-Human Services Survey:** Copyright ©1981 Christina Maslach & Susan E. Jackson.
**MBI-Educators Survey:** Copyright ©1986 Christina Maslach, Susan E. Jackson & Richard L. Schwab.
All rights reserved in all media. Published by Mind Garden, Inc., www.mindgarden.com

For use by Dragana Backovic only. Received from Mind Garden, Inc. on January 27, 2020

Iz razloga što će na ovaj upitnik odgovarati osobe različitih zanimanja , koristi se termin klijenti da označi ljude kojima se obezbeđuje usluga, nega, lečenje ili instrukcija. Kada odgovarate na pitanja molim Vas mislite na ove ljude kao primaoce usluga koje im obezbeđujete , iako možda u svom poslu za njih korisite drugi termin.

Uputstvo: Na sledećim stranama nalaze se 22 tvrdnje vezane za osećanja prema poslu. Molimo Vas da pažljivo pročitate svaku tvrdnju i odlučite da li ste se ikada tako osećali na svom poslu. Ako se nikada niste tako osećali, upištie 0 ( nula) u prostor ispred tvrdnje. Ako ste se tako osećali, naznačite koliko često ste se tako osećali upisujući broj ( od 1 do 6) koji najbolje opisuje učestalost takvog osećanja. Primer je prikazan u daljem tekstu.

PRIMER

Koliko često: 0 nikad
1 nekoliko puta godišnje ili manje
2 jednom mesečno ili manje
3 nekoliko puta mesečno
4 jednom sedmično
5 nekoliko puta sedmično
6 svakodnevno

Koliko često 0-6

1. ___________

Tvrdnja Osećam se depresivno (potišteno) na poslu

Ako se nikada niste osećali depresivno na poslu, upisaćete 0 ( nula) ispod „ koliko često“. Ako ste se retko osećali depresivno na poslu ( nekoliko puta godišnje ili manje), upisaćete 1. Ako je osećanje depresivnosti poprilično učestalo ( nekoliko puta sedmično ali ne svakodnevno), upisaćete broj 5.

MBI- Kjuč za bodovanje upitnika u humanističkim službama i edukativnim zanimanjima

**Lična personalna ostvarenost subskala**

Uputstva: Postavite brojeve sa ovog ključa pored istih brojeva na upitniku. Gledajući samo nezasenčene delove , upišite rezultate u „ koliko često“ kolonu i unesite totalni zbir u prostor „ lična profesionalna ostvarenost“ na dnu upitnika

Kategorizacija: Lična personalna ostvarenost – formular za humanističke službe

| | Učestalost |
|---|---|
| Visoka | 0-31 |
| Umerena | 32-38 |
| Niska | 39 i više |

Interpretira se inverzno od emocionalne iscrpenosti i depersonalizacije

**TA-213 MBI-HSS - Serbian**

**MBI-General Survey:** Copyright ©1996 Wilmar B. Schaufeli, Michael P. Leiter, Christina Maslach & Susan E. Jackson.
**MBI-Human Services Survey:** Copyright ©1981 Christina Maslach & Susan E. Jackson.
**MBI-Educators Survey:** Copyright ©1986 Christina Maslach, Susan E. Jackson & Richard L. Schwab.
All rights reserved in all media. Published by Mind Garden, Inc., www.mindgarden.com

For use by Dragana Backovic only. Received from Mind Garden, Inc. on January 27, 2020

Napomena istraživačima: izveštaji istraživanja koji su koristili MBI-upitnik za humanističke službe i edukativne profesije obično iznose prosečne ocene pre nego totalne zbirove. Da utvrdite prosečnu ocenu za svaku subskalu, podelite ukupan zbir sa brojem odgovorenih upitnika. Skala za ličnu profesionalnu ostvarenost sadrži osam tvrdnji.

MBI- Kjuč za bodovanje upitnika u humanističkim službama i edukativnim zanimanjima

**Subskala depersonalizacije**

Uputstva: Postavite brojeve sa ovog ključa pored istih brojeva na upitniku. Gledajući samo nezasenčene delove , upišite rezultate u „ koliko često" kolonu i unesite totalni zbir u prostor „ depersonalizacija" na dnu upitnika

Kategorizacija: Depersonalizacija – formular za humanističke službe

| | Učestalost |
|---|---|
| Visoka | 13 ili više |
| Umerena | 7-12 |
| Niska | 0-6 |

Napomena istraživačima: izveštaji istraživanja koji su koristili MBI-upitnik za humanističke službe i edukativne profesije obično iznose prosečne ocene pre nego totalne zbirove. Da utvrdite prosečnu ocenu za svaku subskalu, podelite ukupan zbir sa brojem odgovorenih upitnika. Skala za depersonalizaciju sadrži pet tvrdnji.

MBI- Kjuč za bodovanje upitnika u humanističkim službama i edukativnim zanimanjima

**Subskala emocionalne iscrpljenosti**

Uputstva: Postavite brojeve sa ovog ključa pored istih brojeva na upitniku. Gledajući samo nezasenčene delove , upišite rezultate u „ koliko često" kolonu i unesite totalni zbir u prostor „ emocionalna iscrpljenost" na dnu upitnika

Kategorizacija: Emocionalana iscrpljenost – formular za humanističke službe

| | Učestalost |
|---|---|
| Visoka | 27 ili više |
| Umerena | 17-26 |
| Niska | 0-16 |

Napomena istraživačima: izveštaji istraživanja koji su koristili MBI-upitnik za humanističke službe i edukativne profesije obično iznose prosečne ocene pre nego totalne zbirove. Da utvrdite prosečnu ocenu za svaku subskalu, podelite ukupan zbir sa brojem odgovorenih upitnika. Skala za emocionalnu iscrpenost sadrži devet tvrdnji.



**MBI-General Survey:** Copyright ©1996 Wilmar B. Schaufeli, Michael P. Leiter, Christina Maslach & Susan E. Jackson.
**MBI-Human Services Survey:** Copyright ©1981 Christina Maslach & Susan E. Jackson.
**MBI-Educators Survey:** Copyright ©1986 Christina Maslach, Susan E. Jackson & Richard L. Schwab.
All rights reserved in all media. Published by Mind Garden, Inc., www.mindgarden.com

For use by Dragana Backovic only. Received from Mind Garden, Inc. on January 27, 2020

# MBI-Human Services Survey

*Cilj ovog upitnika je da se otkrije kako različite osobe u uslužnim delatnostima ili profesionalci koji pomažu ljudima, vide svoj posao ili ljude, sa kojima blisko sarađuju.*

Zato što će ljudi različitih zanimanjima popunjavati upitnik, u primeni je izraz „korisnik" što se odnosi na ljude kojima pružate svoje usluge, negu, tretman ili uputstva. Prilikom popunjavanja upitnika, molim Vas da o tim ljudima razmišljate kao o korisnicima Vaših usluga, i pored toga što u svom radu možete koristiti drugi termin.

Uputstva: U produžetku su 22 izjave ličnih osećanja povezanih sa poslom. Molim Vas da svaku izjavu pročitate pažljivo odlučite da li se ikada osećate tako, u vezi svog posla. Ako nikada niste imali taj osećaj, upišite broj „0" (nula) u prostor ispred izjave. Ako ste imali taj osećaj, pokažite koliko često se tako osećate upisivanjem broja (od 1 do 6) koji naj bolje opisuje koliko često se tako osećate.
Jedan primer je prikazan naniže:

**Primer:**

| **Koliko često (0-6):** | **0** | **1** | **2** | **3** | **4** | **5** | **6** |
|---|---|---|---|---|---|---|---|
| | Nikad | Nekoliko puta godišnje ili ređe | Jednom mesečno ili ređe | Nekoliko puta mesečno | Jednom nedeljno | Nekoliko puta nedeljno | Svaki dan |

**Koliko često (0-6)** **Izjava:**

1. ___________ Osećam se depresivno na poslu.

Ako se nikada ne osećate depresivno na poslu, onda bi ste upisali broj „0" (nula) ispod naslova „Koliko često". Ako se retko osećate depresivno na poslu (nekoliko puta godišnje ili manje) onda bi ste upisali broj „1". Ako je Vaš osećaj depresije prilično čest (nekoliko puta nedeljno ali ne svakodnevno), onda biste upisali broj „5".



**MBI-General Survey:** Copyright ©1996 Wilmar B. Schaufeli, Michael P. Leiter, Christina Maslach & Susan E. Jackson.
**MBI-Human Services Survey:** Copyright ©1981 Christina Maslach & Susan E. Jackson.
**MBI-Educators Survey:** Copyright ©1986 Christina Maslach, Susan E. Jackson & Richard L. Schwab.
All rights reserved in all media. Published by Mind Garden, Inc., www.mindgarden.com

For use by Dragana Backovic only. Received from Mind Garden, Inc. on January 27, 2020

| Koliko često: | 0 | 1 | 2 | 3 | 4 | 5 | 6 |
|---|---|---|---|---|---|---|---|
| | Nikad | Nekoliko puta godišnje ili ređe | Jednom mesečno ili ređe | Nekoliko puta mesečno | Jednom nedeljno | Nekoliko puta nedeljno | Svaki dan |

**Koliko često 0-6** **Izjave:**

1. _________ Osećam se emocionalno iscrpljeno zbog posla.
2. _________ Osećam se iskorišćeno na kraju radnog dana.
3. _________ Osećam se umorno kada ujutru ustanem i moram da se suočim sa još jednim danom na poslu.
4. _________ Mogu lako da razumem kako se moji korisnici osećaju.
5. _________ Imam osećaj da se prema nekim korisnicima ophodim kao da su bezlični objekti.
6. _________ Celodnevni rad sa ljudima je zaista napor za mene.
7. _________ Vrlo efikasno rešavam probleme svojih korisnika.
8. _________ Osećam da izgaram zbog posla.
9. _________ Osećam da kroz svoj posao pozitivno utičem na živote drugih.
10. _________ Od kad imam ovaj posao postao/la sam bezosećajan/a prema ljudima.
11. _________ Brinem se da me ovaj posao emocionalno otupljuje.
12. _________ Osećam se veoma snažno.
13. _________ Osećam da sam isfrustriran/a poslom.
14. _________ Imam osećaj da previše radim na poslu.
15. _________ U suštini mi je svejedno šta se dešava sa nekim korisnicima.
16. _________ Rad sa ljudima je previše stresan za mene.
17. _________ Ja mogu lako da stvorim opuštenu atmosferu sa svojim korisnicima.
18. _________ Osećam zadovoljstvo nakon rada sa korisnicima.
19. _________ Ostvario/la sam mnogo stvari vrednih truda na ovom poslu.
20. _________ Osećam da sam na izmaku snaga.
21. _________ U svom radu, emotivne probleme rešavam vrlo smireno.
22. _________ Imam osećaj da me korisnici krive za neke od svojih problema.

(Za administrativnu primenu)

**EE:** _______ cat:_______ **DP:** _______ cat:_______ **PA:** _______ cat:_______

**TA-337 – MBI-HSS - Serbian**

**MBI-General Survey:** Copyright ©1996 Wilmar B. Schaufeli, Michael P. Leiter, Christina Maslach & Susan E. Jackson.
**MBI-Human Services Survey:** Copyright ©1981 Christina Maslach & Susan E. Jackson.
**MBI-Educators Survey:** Copyright ©1986 Christina Maslach, Susan E. Jackson & Richard L. Schwab.
All rights reserved in all media. Published by Mind Garden, Inc., www.mindgarden.com

For use by Dragana Backovic only. Received from Mind Garden, Inc. on January 27, 2020

# MBI-ES

| 0 | 1 | 2 | 3 | 4 | 5 | 6 |
|---|---|---|---|---|---|---|
| nikad | nekoliko puta godišnje | jednom mesečno ili manje | nekoliko puta mesečno | jednom nedeljno | nekoliko puta nedeljno | svakog dana |

1. Osećam se emocionalno iscrpljeno zbog svog posla
2. Osećam se potrošeno na kraju radnog dana
3. Umoran sam već kad se probudim i moram da se suočim sa još jednim radnim danom
4. S lakoćom razumem kako se moji učenici osećaju
5. Nekada imam bezličan, mehanički odnos prema učenicima
6. Svakodnevni rad sa ljudima je zaista naporan za mene
7. Veoma efikasno rešavam probleme mojih učenika
8. Osećam se "sagorelo" zbog svog posla
9. Osećam da, svojim radom, pozitivno utičem na živote drugih ljudi
10. Postao sam bezosećajan ( "krut") prema ljudima od kada se bavim ovim poslom
11. Mislim da me ovaj posao emocionalno zatupljuje
12. Osećam da sam pun energije
13. Isfrustriran sam svojim poslom
14. Osećam da mi je na poslu suviše napomo
15. Nije mi stalo do pojedinih učenika
16. Direktan kontakt s ljudima na poslu je suviše stresan za mene
17. S lakoćom stvaram opuštenu atmosferu pri radu s učenicima
18. Raspoložen sam nakon rada sa mojim učenicima
19. Dobio sam mnogo toga vrednog od ovog posla
20. Osećam da sam na granici izdržljivosti
21. Mogu mirno da se nosim s emocionalnim problemima na svom poslu
22. Osećam da me učenici krive zbog svojih problema


**MBI-General Survey:** Copyright ©1996 Wilmar B. Schaufeli, Michael P. Leiter, Christina Maslach & Susan E. Jackson.
**MBI-Human Services Survey:** Copyright ©1981 Christina Maslach & Susan E. Jackson.
**MBI-Educators Survey:** Copyright ©1986 Christina Maslach, Susan E. Jackson & Richard L. Schwab.
All rights reserved in all media. Published by Mind Garden, Inc., www.mindgarden.com

For use by  Dragana Backovic only. Received from Mind Garden, Inc. on January 27, 2020

**For Dissertation and Thesis Appendices:**

You cannot include an entire instrument in your thesis or dissertation, however you can use up to three sample items. Academic committees understand the requirements of copyright and are satisfied with sample items for appendices and tables. For customers needing permission to reproduce three sample items in a proposal, thesis, or dissertation the following page includes the permission form and reference information needed to satisfy the requirements of an academic committee.

**Putting Mind Garden Instruments on the Web:**

If your research uses a Web form, you will need to meet Mind Garden's requirements by following the procedure described at http://www.mindgarden.com/how.htm#instrumentweb.

**All Other Special Reproductions:**

For any other special purposes requiring permissions for reproduction of this instrument, please contact info@mindgarden.com.

**MBI-General Survey:** Copyright ©1996 Wilmar B. Schaufeli, Michael P. Leiter, Christina Maslach & Susan E. Jackson.
**MBI-Human Services Survey:** Copyright ©1981 Christina Maslach & Susan E. Jackson.
**MBI-Educators Survey:** Copyright ©1986 Christina Maslach, Susan E. Jackson & Richard L. Schwab.
All rights reserved in all media. Published by Mind Garden, Inc., www.mindgarden.com

For use by Dragana Backovic only. Received from Mind Garden, Inc. on January 27, 2020

mind garden

www.mindgarden.com

To whom it may concern,

This letter is to grant permission for the above named person to use the following copyright material for his/her thesis or dissertation research:

Instrument: ***Maslach Burnout Inventory, Forms: General Survey, Human Services Survey & Educators Survey***

## Copyrights:

**MBI-General Survey (MBI-GS)**: Copyright ©1996 Wilmar B. Schaufeli, Michael P. Leiter, Christina Maslach & Susan E. Jackson. All rights reserved in all media. Published by Mind Garden, Inc., www.mindgarden.com

**MBI-Human Services Survey (MBI-HSS)**: Copyright ©1981 Christina Maslach & Susan E. Jackson. All rights reserved in all media. Published by Mind Garden, Inc., www.mindgarden.com

**MBI-Educators Survey (MBI-ES)**: Copyright ©1986 Christina Maslach, Susan E. Jackson & Richard L. Schwab. All rights reserved in all media. Published by Mind Garden, Inc., www.mindgarden.com

Three sample items from a single form of this instrument may be reproduced for inclusion in a proposal, thesis, or dissertation.

The entire instrument may not be included or reproduced at any time in any published material.

Sincerely,

Robert Most
Mind Garden, Inc.
www.mindgarden.com

**MBI-General Survey**: Copyright ©1996 Wilmar B. Schaufeli, Michael P. Leiter, Christina Maslach & Susan E. Jackson.
**MBI-Human Services Survey**: Copyright ©1981 Christina Maslach & Susan E. Jackson.
**MBI-Educators Survey**: Copyright ©1986 Christina Maslach, Susan E. Jackson & Richard L. Schwab.
All rights reserved in all media. Published by Mind Garden, Inc., www.mindgarden.com

Prilog 3. Covid stress skala

## COVID-19 stres skala

Navedena pitanja se odnose na različite vrste briga koje ste možda iskusili u **poslednjih sedam dana.** U navedenim iskazima / rečenicama kada se spominje virus odnosi se na COVID-19.

| | Ne uopšte | Malo | Umereno | Veoma | Izraženo |
|---|---|---|---|---|---|
| 1. Brinem se da ne dobijem virus | 0 | 1 | 2 | 3 | 4 |
| 2. Brinem se da ne mogu da zaštitim svoju porodicu od virusa | 0 | 1 | 2 | 3 | 4 |
| 3. Brinem se da naš zdravstveni sistem neće moći da zaštiti osobe koje volim | 0 | 1 | 2 | 3 | 4 |
| 4. Brinem se da naš zdravstveni sistem ne može da me zaštiti od virusa | 0 | 1 | 2 | 3 | 4 |
| 5. Brinem se da osnovna higijena (npr. pranje ruku) nije dovoljna da me zaštiti od virusa | 0 | 1 | 2 | 3 | 4 |
| 6. Brinem se da socijalno distanciranje nije dovoljno da me zaštiti od virusa | 0 | 1 | 2 | 3 | 4 |
| 7. Brinem se da će prodavnice ostati bez hrane | 0 | 1 | 2 | 3 | 4 |
| 8. Brinem se da će se prodavnice zatvoriti | 0 | 1 | 2 | 3 | 4 |
| 9. Brinem se da će prodavnice ostati bez sredstava za čišćenje i dezinfekciju | 0 | 1 | 2 | 3 | 4 |
| 10. Brinem se da će apoteke / prodavnice ostati bez lekova protiv prehlade ili gripa | 0 | 1 | 2 | 3 | 4 |
| 11. Brinem se da će prodavnice ostati bez vode | 0 | 1 | 2 | 3 | 4 |
| 12. Brinem se da će apoteke ostati bez lekova koji se kupuju na recept | 0 | 1 | 2 | 3 | 4 |
| 13. Brinem se da stranci šire virus u mojoj zemlji | 0 | 1 | 2 | 3 | 4 |
| 14. Kada bih išao/la u restoran specijalizovan za inostranu kuhinju brinuo/la bih se da ne dobijem virus | 0 | 1 | 2 | 3 | 4 |
| 15. Brinem se da dođem u kontakt sa strancima jer oni možda imaju virus | 0 | 1 | 2 | 3 | 4 |
| 16. Kada bih upoznao/la osobu iz inostranstva brinuo/la bih se da ona možda ima virus | 0 | 1 | 2 | 3 | 4 |
| 17. Kada bih bio/la u liftu sa grupom stranaca brinuo/la bih se da su oni možda zaraženi virusom | 0 | 1 | 2 | 3 | 4 |
| 18. Brinem se da stranci šire virus zato što ne vode računa o higijeni kao mi | 0 | 1 | 2 | 3 | 4 |
| 19. Brinem se da ću ako dodirnem nešto na javnom mestu (npr. gelender, kvaku) dobiti virus | 0 | 1 | 2 | 3 | 4 |
| 20. Brinem se da ću ako se neko zakašlje ili kine u mojoj blizini dobiti virus | 0 | 1 | 2 | 3 | 4 |
| 21. Brinem se da će ljudi iz moje okoline da me zaraze virusom | 0 | 1 | 2 | 3 | 4 |
| 22. Brinem se da uzimam kusur prilikom plaćanja u kešu | 0 | 1 | 2 | 3 | 4 |

| | | | | | |
|---|---|---|---|---|---|
| 23. Brinem se da mogu da dobijem virus prilikom rukovanja sa novcem ili korišćenjem bankomata | 0 | 1 | 2 | 3 | 4 |
| 24. Brinem se da poštar ne zagadi moju poštu | 0 | 1 | 2 | 3 | 4 |

Molimo Vas da pročitate svaku rečenicu i označite / zaokružite koliko često ste iskusili svaki od navedenih problema u **poslednjih sedam dana.**

| | Nikad | Retko | Ponekad | Često | Skoro stalno |
|---|---|---|---|---|---|
| 25. Teško sam se koncentrisao/la jer sam razmišljao/la o virusu | 0 | 1 | 2 | 3 | 4 |
| 26. Uznemirujuće slike o virusu mi prolaze kroz glavu bez moje volje | 0 | 1 | 2 | 3 | 4 |
| 27. Loše sam spavao/la jer sam se brinuo/la o virusu | 0 | 1 | 2 | 3 | 4 |
| 28. Razmišljao/la sam o virusu i kad nisam hteo/la | 0 | 1 | 2 | 3 | 4 |
| 29. Stvari koje me podsećaju na virus u meni izazivaju fizičke reakcije kao što su preznojavanje ili lupanje srca | 0 | 1 | 2 | 3 | 4 |
| 30. Imao/la sam noćne more (košmare) o virusu | 0 | 1 | 2 | 3 | 4 |

Sledeći iskazi / rečenice ispituju ponašanje. **Tokom poslednjih sedam dana,** koliko često ste radili navedeno usled zabrinutosti zbog COVID-19?

| | Nikad | Retko | Ponekad | Često | Skoro stalno |
|---|---|---|---|---|---|
| 31. Pretraživao/la internet o načinima lečenja COVID-19 | 0 | 1 | 2 | 3 | 4 |
| 32. Tražio savet od zdravstvenih radnika (npr. doktora ili farmaceuta) o COVID-19 | 0 | 1 | 2 | 3 | 4 |
| 33. Gledao/la YouTube video snimke o COVID-19 | 0 | 1 | 2 | 3 | 4 |
| 34. Pregledao/la sam se u potrazi za znacima infekcije (npr. merenje temperature) | 0 | 1 | 2 | 3 | 4 |
| 35. Tražilo/la sam podršku od prijatelja ili porodice zbog COVID-19 | 0 | 1 | 2 | 3 | 4 |
| 36. Proveravao/la sam šta piše u objavama na društvenim mrežama u vezi sa COVID-19 | 0 | 1 | 2 | 3 | 4 |

Prilog 4. AIS skala - ATENSKA SKALA ZA NESANICU

# ATENSKA SKALA ZA NESANICU

*Svrha ove skale je da zabilježi vašu vlastitu procjenu poteškoća sa snom koje ste možda iskusili. Molimo, obilježite (zaokruživanjem odabranog broja) u dole navedenim redovima brojeve koji po vašoj procjeni odgovaraju problemima koji su vam se desili najmanje tri puta sedmično unutar proteklih mjesec dana.*

1. PROBLEM DA ZASPITE *(vrijeme potrebno da zaspite nakon gašenja svjetla)*

| 0 | 1 | 2 | 3 |
|---|---|---|---|
| Bez problema | Malo odloženo | Značajno odloženo | Vrlo dugo odloženo ili uopće niste zaspali |

2. BUDJENJA TOKOM SNA

| 0 | 1 | 2 | 3 |
|---|---|---|---|
| Bez budjenja | Neznatan problem | Znatan problem | Ozbiljan problem ili uopće niste zaspali |

3. KONAČNO BUDJENJE RANIJE NEGO ŠTO STE ŽELJELI

| 0 | 1 | 2 | 3 |
|---|---|---|---|
| Ne ranije | Malo ranije | Znatno ranije | Mnogo ranije ili uopće niste zaspali |

4. UKUPNO VRIJEME SPAVANJA

| 0 | 1 | 2 | 3 |
|---|---|---|---|
| Dovoljno | Lagano nedovoljno | Znatno nedovoljno | Vrlo nedovoljno ili uopće nist zaspali |

5. SVEUKUPNI KVALITET SNA *(bez obzira koliko dugo ste spavali)*

| 0 | 1 | 2 | 3 |
|---|---|---|---|
| Zadovoljavajući | Malo nezadovoljavajući | Značajno nezadovoljavajuć | Vrlo nezadovoljavajući ili uopće niste spavali |

6. KAKO SE OSJEĆATE TOKOM DANA

| 0 | 1 | 2 | 3 |
|---|---|---|---|
| Normalno | Malo oslabljeno | Znatno oslabljeno | Jako oslabljeno |

7. FUNKCIONISANJE (FIZICKO I DUŠEVNO) TOKOM DANA

| 0 | 1 | 2 | 3 |
|---|---|---|---|
| Normalno | Malo slabije | Znatno slabije | Jako slabo |

8. POSPANOST TOKOM DANA

| 0 | 1 | 2 | 3 |
|---|---|---|---|
| Ne postoji | Mala pospanost | Značajna pospanost | Jaka pospanost |

Prilog 5. DASS skala - Skala depresivnosti, anksioznosti i stresa

| 0.Uopšte se ne odnosi na mene;<br>1.Primjereno na mene do neke mjere ili neko vrieme;<br>2.Primijenjeno u znatnoj mjeri ili dobar dio vremena;<br>3 Primijenjeno na mene puno ili većini vremena. | Komponenta | | | |
|---|---|---|---|---|
| | 0 | 1 | 2 | 3 |
| 1.Bilo mi je teško da se smirim | | | | |
| 2.Primetio sam da mi se suše usta. | | | | |
| 3.Nisam imao nikakvo lepo osjecanje. | | | | |
| 4.Imao sam poteskoća sa disanjem. | | | | |
| 5.Primjetio sam da mi je teško da ostvarim incijativu i započnem bilo šta. | | | | |
| 6.Preterano reaguem u nekim situaciama. | | | | |
| 7.sjetio sam da se tresem (npr.tresle su mi se ruke ). | | | | |
| 8. Primetio sam da koristim dosta „nervozne energije”. | | | | |
| 9. Bojao sam se situacija u kojima bih mogao da se uspaničim i napravim budalu od sebe. | | | | |
| 10. Osećao sam da nemam čemu da se nadam. | | | | |
| 11. Primetio sam da se nerviram. | | | | |
| 12. Teško mi je da se opustim. | | | | |
| 13. Osećao sam se tužno i jadno. | | | | |
| 14. Nerviralo me je kada me nešto prekida u onome što radim. | | | | |
| 15. Osećao sam da sam blizu panike. | | | | |
| 16. Ništa nije moglo da me zainteresuje. | | | | |
| 17. Oseao sam se da kao osoba ne vredim mnogo | | | | |
| 18. Bio sam jako osetljiv. | | | | |
| 19. Osetio sam rad srca iako se nisam fizički zamorio. | | | | |
| 20. Osećao sam se uplašeno bez razloga. | | | | |
| 21. Osećao sam da je život besmislen. | | | | |
